# HPV Knowledge, Vaccination Uptake, and Salivary Diagnostics Among Dental Students in Romania

**DOI:** 10.3390/vaccines13060658

**Published:** 2025-06-19

**Authors:** Sergiu Baranga, Doina Chioran, Octavia Balean, Ramona Dumitrescu, Roxana Popescu, Daniela Jumanca, Roxana Oancea, Ruxandra Sava-Rosianu, Vanessa Bolchis, Atena Galuscan

**Affiliations:** 1 Doctoral School, “Victor Babes” University of Medicine and Pharmacy, Eftimie Murgu Sq. No. 2, 300041 Timisoara, Romania; sergiu.baranga@umft.ro; 2 Department of Anesthesiology and Oral Surgery, “Victor Babes” University of Medicine and Pharmacy, Eftimie Murgu Sq. No. 2, 300041 Timisoara, Romania; chioran.doina@umft.ro; 3 Translational and Experimental Clinical Research Centre in Oral Health, Department of Preventive, Community Dentistry and Oral Health, University of Medicine and Pharmacy “Victor Babes”, 300040 Timisoara, Romania; jumanca.daniela@umft.ro (D.J.); roancea@umft.ro (R.O.); sava-rosianu.ruxandra@umft.ro (R.S.-R.); vanessa.bolchis@umft.ro (V.B.); galuscan.atena@umft.ro (A.G.); 4 Clinic of Preventive, Community Dentistry and Oral Health, “Victor Babes” University of Medicine and Pharmacy, Eftimie Murgu Sq. No. 2, 300041 Timisoara, Romania; 5 ANAPATMOL Research Center, Faculty of Medicine, “Victor Babes” University of Medicine and Pharmacy Timisoara, 300041 Timisoara, Romania; popescu.roxana@umft.ro; 6 Department of Cell and Molecular Biology, “Victor Babes” University of Medicine and Pharmacy Timisoara, 300041 Timisoara, Romania

**Keywords:** HPV awareness, vaccination uptake, salivary screening, dental students, oral cancer

## Abstract

**Background:** Human papillomavirus (HPV) is a key cause of cervical and oropharyngeal cancers. Despite available vaccines, uptake remains low in Romania due to limited awareness and hesitancy. This study assessed HPV knowledge, vaccination status, and the presence of high-risk strains (16 and 18) in the saliva of dental students from Victor Babeș University in Timișoara. **Methods:** A cross-sectional study was conducted between February and March 2024, enrolling 199 dental students. Participants completed a 15-item questionnaire addressing HPV-related knowledge, vaccination status, lifestyle factors, and health history. Saliva samples were collected and analyzed using real-time PCR for the detection of HPV types 16 and 18. Logistic regression analysis was employed to identify predictors of vaccination uptake. **Results:** Only 10.6% of participants had received the HPV vaccine, although 96.9% acknowledged its safety and efficacy. Awareness was higher among females (88.1%) than males (84.3%), and vaccination rates were significantly greater among students under 25 years old (*p* = 0.0312). A total of 16.6% reported the presence of papillomas or warts. HPV DNA was detected in 10% of saliva samples. **Conclusions:** Although awareness of HPV was high, vaccination rates remained low, revealing a gap between knowledge and preventive action. Saliva-based screening shows promise as a non-invasive diagnostic tool, and integrating targeted education and advocacy into dental curricula may enhance public health outcomes in Romania.

## 1. Introduction

### 1.1. Introduction

Human papillomavirus (HPV) is one of the most widespread viral infections, significantly contributing to cancer development later in life. It is estimated that HPV is responsible for approximately 570,000 new cancer cases annually in females and 60,000 in males [1]. This infection is associated with multiple malignancies, including cervical, vaginal, and vulvar cancers in females, penile cancer in males, and anal and oropharyngeal cancers in both sexes [1,2]. The burden of HPV-related cancers has led the World Health Organization (WHO) to establish a global initiative aimed at eliminating these malignancies, particularly cervical cancer, by 2030 [2].

Although HPV is primarily known for its role in cervical cancer, its association with other cancers, particularly those of the head and neck, has gained increasing attention. The persistence of HPV infection is a key factor in cancer development, with high-risk strains such as HPV 16 and HPV 18 accounting for the majority of HPV-associated malignancies [3,4]. Over 200 HPV types have been identified, with 14 considered high-risk [5]. Although cervical cancer benefits from effective screening tools, standardized methods for detecting other HPV-related malignancies, such as oropharyngeal cancer, remain limited [4,6].

The increasing incidence of HPV-positive oropharyngeal cancers (OPCs) has contributed to a shift in the epidemiology of head and neck malignancies. Head and neck squamous cell carcinoma (HNSCC) is a category of cancers affecting the oral cavity, throat, and larynx, with approximately 453,000 deaths attributed to HNSCC globally. Traditionally, alcohol consumption and tobacco use were considered the primary risk factors for HNSCC, but the emergence of HPV-positive OPCs has introduced a new dimension to the disease’s etiology [7]. Recent studies have revealed that HPV-positive oropharyngeal cancers are increasingly diagnosed in younger male patients with little or no history of tobacco or alcohol use, but with a high number of lifetime sexual partners—especially involving oral sex. For instance, individuals with more than nine lifetime sexual partners have a 34-fold higher risk of developing oropharyngeal cancer. These cancers are most commonly caused by HPV types 16 and 18 and tend to have better treatment responses and survival rates compared to their HPV-negative counterparts [8]. The evolving patterns of tobacco use and the increasing prevalence of HPV-related OPCs have significantly altered the epidemiological landscape of head and neck cancers, necessitating a reevaluation of current prevention and treatment strategies [9]. Despite the fact that HPV vaccines are not yet specifically approved for preventing OPC, the molecular and epidemiological association strongly supports the role of immunization in reducing this burden. Therefore, increasing awareness and vaccine coverage among young adults, particularly those in healthcare training, remains a critical step in cancer prevention [10].

Given the growing prevalence of HPV-associated OPCs, healthcare professionals, including dentists, play a crucial role in HPV prevention and patient education. Dental professionals are in a unique position to raise awareness about the risks associated with HPV infection, encourage vaccination, and discuss the links between HPV and OPCs. As future healthcare providers, dental students must acquire the necessary knowledge, develop a positive attitude toward HPV vaccination, and enhance their communication skills to effectively counsel patients on HPV prevention strategies [11]. Recent European studies [12,13] reveal that dental students often lack adequate training in HPV-related communication, particularly on sexual health topics. This suggests a broader need for curriculum reform and targeted training in dental education internationally. Strengthening the role of dentists in HPV prevention can contribute to a broader public health effort aimed at reducing HPV-related cancer incidence. University education plays a key role in developing both professional competencies and public health awareness among future dental practitioners. Training should also foster interdisciplinary skills such as infection control, patient communication, and health promotion to meet the evolving demands of dental care. To better understand the current state of knowledge and prevention efforts, a review of relevant literature is presented below.

### 1.2. Literature Review

In addition to vaccination and traditional screening methods, saliva-based diagnostics have emerged as a promising non-invasive tool for detecting HPV-related malignancies [14]. In the United States, HPV is estimated to be responsible for approximately 70% PA of oropharyngeal cancers (OPCs), with cases now surpassing those of HPV-related cervical cancer [4]. Saliva is a hypotonic biofluid that contains biochemical markers capable of providing insights into prognosis, laboratory diagnostics, and patient monitoring. The ease of collection, non-invasiveness, and safety of saliva sampling make it an ideal biofluid for diagnostic applications. Given its ability to reflect genomic, proteomic, transcriptomic, epigenomic, metabolomic, microbiomic, and pathological changes in the head and neck region, saliva has significant potential as a liquid biopsy for early disease detection [15,16].

Beyond its diagnostic potential, HPV vaccination represents a groundbreaking advancement in cancer prevention. As the first vaccine developed to prevent a cancer, HPV vaccination has been widely adopted in Europe. Initially, it was available only for females, but some countries, including Austria and Italy, have extended vaccination programs to males. Expanding vaccination coverage to boys not only protects them from anal cancers and genital warts but also contributes to herd immunity, reducing HPV transmission within the population. However, the WHO continues to prioritize cervical cancer prevention, emphasizing the importance of vaccinating young females before they become sexually active [17].

Although the HPV vaccine has been primarily associated with cervical cancer prevention, emerging research suggests that it may also offer protection against other HPV-related malignancies, including oral and oropharyngeal cancers. Given the growing burden of HPV-related OPCs, expanding research on the potential role of HPV vaccination in preventing these cancers is of paramount importance [18]. Integrating vaccination, early screening, and novel diagnostic approaches such as saliva-based testing can collectively contribute to reducing the incidence of HPV-related malignancies. Although the HPV vaccine is not currently licensed specifically for the prevention of oropharyngeal cancer, the biological plausibility and overlap in viral etiology support its use as a preventive strategy. Official recommendations by organizations such as the CDC (Centers for Disease Control and Prevention) and WHO increasingly advocate for gender-neutral vaccination strategies to address this growing burden [19].

The Romanian HPV vaccination experience began in 2008 but failed to gain widespread acceptance until 2020, primarily due to a low level of public awareness and hesitancy regarding the vaccine [20]. In response to the high burden of HPV-related diseases, the Romanian Ministry of Health launched a renewed HPV vaccination campaign in 2020, continuously adapting it to the needs of the population [21]. Cervical cancer remains the second most common cancer among Romanian women aged 15 to 44 years, highlighting the urgent need for effective prevention strategies [22].

In May 2025, Romania adopted new legislation to officially integrate HPV vaccination into the National Immunization Program (NIP), extending free access to all girls and boys aged 11 to 26 years [23]. This expansion, delivered through family physicians, represents a significant milestone in national public health efforts to reduce HPV-related cancers. The move aligns with the World Health Organization’s call for 90% HPV vaccine coverage by 2030 and responds to Romania’s persistently high cervical cancer mortality rates—among the highest in the European Union [24]. Over 1800 women die annually from cervical cancer in Romania, despite its being a largely preventable disease. The new policy follows the previous campaign-based system, where free vaccination was available only upon request for girls and boys aged 11–18, with 50% reimbursement for adults up to age 45 [25]. By adopting a comprehensive, gender-neutral vaccination approach, Romania joins other countries such as Austria, Italy, the UK, Canada, and Australia in aiming to curb HPV transmission and prevent both cervical and non-cervical HPV-related malignancies. Currently, routine HPV vaccination in Romania is recommended starting at age 11 (with the possibility of initiation at age 9) and up to age 26. For adults aged 27 to 45, vaccination may be considered based on individual benefits [26]. However, parental consent is required for individuals under 18, as HPV vaccination is not included in the National Vaccination Program. Legally, family doctors are responsible for administering HPV vaccines, including access to free doses provided by the Ministry of Health upon request. The vaccine is recommended for both girls and boys, emphasizing its role in reducing HPV transmission and associated cancers. Despite the availability of the vaccine, public attitudes toward HPV vaccination in Romania remain a significant barrier to its success.

In Romania, vaccine hesitancy remains a major barrier to HPV immunization efforts, including among university students. Several studies have demonstrated that informational gaps, coupled with psychological and sociocultural factors, contribute significantly to low vaccine uptake. For example, Voidăzan et al. found that although awareness of HPV was relatively high among medical students, misconceptions about transmission, vaccine safety, and long-term effects persisted, especially among preclinical students [27]. Furthermore, Diaconescu et al. reported that emotional variables—such as external health locus of control and behavioral disengagement—were stronger predictors of hesitancy than knowledge alone [28]. These findings suggest that addressing hesitancy requires more than awareness campaigns and should include tailored interventions that also target attitudes, trust and emotional readiness to vaccinate. Studies indicate that a lack of knowledge or misinformation has been a major factor in parents’ reluctance to vaccinate their children [29,30,31,32].

Although scientific evidence strongly supports HPV vaccination as an effective tool in preventing several types of cancer, particularly cervical cancer, previous HPV vaccination campaigns in Romania have largely failed [33].

Given these challenges, our study aimed to assess the level of awareness, acceptance, and knowledge regarding HPV infection and vaccination among Romanian dental students. In addition, we evaluated the presence of high-risk HPV types 16 and 18 in saliva samples through real-time PCR, to explore the feasibility of saliva-based diagnostics as a non-invasive screening method among asymptomatic young adults. By integrating questionnaire-based insights with molecular testing, the study further aimed to identify possible associations between HPV status, vaccination uptake, and awareness levels, offering a multifaceted view of HPV prevention in a future healthcare workforce.

## 2. Materials and Methods

### 2.1. Study Participants

This cross-sectional study was conducted at the Translational and Experimental Clinical Research Center in Oral Health, within the Clinic of Preventive, Community Dentistry, and Oral Health at the University of Medicine and Pharmacy “Victor Babes,” Timișoara, Romania. The study took place between February and March 2024 and included 199 young adults, all dental medicine students enrolled in the second, fifth, and sixth years of study. Dental education in Romania is a 6-year integrated undergraduate program aligned with EU directives, combining preclinical coursework and clinical training. Upon graduation, students earn a Doctor of Dental Medicine (DMD) degree, qualifying them to practice general dentistry. Postgraduate specialization, regulated by the Ministry of Health, is available through competitive national residency programs lasting three years. Independent practice requires registration with the Romanian College of Dentists, the national regulatory authority.

To ensure adequate representation of the target population, a sample size calculation was performed prior to data collection. Using the finite population correction formula for proportions and considering an estimated total of 600 dental students enrolled in the Romanian section of the faculty, the minimum required sample size was calculated to be 234 participants. The parameters applied included a 95% confidence level, a 5% margin of error, and an assumed response distribution of 50% to account for maximum variability. Although the final sample comprised 199 students, this number represents over 85% of the estimated target and remains within an acceptable range for a cross-sectional observational study conducted under voluntary participation conditions [34,35].

Participants completed a 15-item questionnaire assessing their knowledge about human papillomavirus (HPV), vaccination status, systemic conditions, chronic inflammatory diseases, and smoking habits. Information regarding HPV vaccination status was self-reported by participants through the questionnaire, indicating whether they had received the vaccine or not. The questionnaire was adapted from previously validated tools used in similar studies assessing HPV knowledge among medical and dental students [8,36]. It was reviewed by three dental public health experts for clarity, content validity, and cultural relevance. A pilot version was tested on 15 students to ensure understanding and face validity. Minor linguistic and structural adjustments were made based on this feedback.

Students were divided into three main groups: 58 second-year students, 59 fifth-year students, and 82 sixth-year students. These specific year groups were selected using a convenience sampling approach to ensure a representative mix of junior and senior students. This selection was deliberate to reflect the progression through distinct stages of dental education. Second-year students represent the preclinical phase, primarily focused on theoretical foundations. Fifth-year students are typically immersed in clinical rotations, while sixth-year students are transitioning toward graduation and independent professional practice. This academic stratification enabled a comparative analysis of HPV-related knowledge and attitudes across different levels of training.

Recruitment was conducted during scheduled university sessions, and all eligible students present were invited to participate on a voluntary basis.

Information about the study was provided in person by trained members of the research team during regular academic courses. Participation was entirely voluntary, and no financial or academic incentives were offered. Students who agreed to participate signed a written informed consent form and were included only if they completed both the questionnaire and the saliva sample collection.

The primary variables of the study were HPV vaccination status and knowledge about HPV. The independent variables included the year of study and immunization status. For the purpose of this study, junior dental students were defined as those in the second year, while senior students included those in the fifth and sixth years. Additionally, the study aimed to assess students’ opinions and attitudes toward HPV prevention measures, as well as their confidence in clinical examinations for cancer detection.

For the purpose of age-based comparisons, participants were classified into two groups: those under the age of 25 (152 students, 76.4%) and those aged 25 or older (47 students, 23.6%). This categorization aligns with the structure of Romanian dental education, where students aged 25 and above are generally in the final years of their undergraduate program or are preparing for postgraduate specialization examinations. Although the average age of respondents was 23.5 years, those in the older age group are more likely to exhibit higher levels of clinical maturity and autonomy in health-related decision-making. These factors may influence their perspectives and behaviors concerning HPV prevention and vaccination.

This study complied with the ethical guidelines set forth in the World Medical Association’s Declaration of Helsinki (1964). Approval was granted by the Ethical Committee of the University of Medicine and Pharmacy “Victor Babes”, Timisoara, Romania (No. 15/15.01.2024). Participation was entirely voluntary, and all participants provided informed consent by reading and signing the consent form. Inclusion criteria comprised dental students enrolled in the 2nd, 5th, or 6th year who provided informed consent. Exclusion criteria included refusal to provide saliva samples or incomplete questionnaire responses.

In addition to assessing HPV-related knowledge, attitudes, and vaccination behaviors through a structured questionnaire, the study included a complementary biological investigation component. Specifically, participants were also subjected to salivary screening aimed at detecting HPV genotypes. This methodological integration allowed for a more robust and multidimensional analysis, facilitating the correlation between self-reported awareness and actual infection status. The inclusion of molecular testing reflects the study’s broader objective of bridging the gap between perceived and biological risk among future dental professionals.

### 2.2. Saliva Collection

Saliva samples were collected from consenting participants using a small, pre-labeled sterile Eppendorf collection tube. To standardize conditions and minimize contamination, participants were instructed to refrain from consuming food and beverages (except water) for at least two hours prior to sample collection. Additionally, smokers were advised to abstain from smoking for at least one hour before sampling, and oral hygiene procedures were avoided during the same period [37].

To prevent DNA degradation, all samples were stored at low temperatures immediately after collection. Samples were immediately stored at 4 °C and subsequently frozen at −80 °C until DNA extraction. All samples were coded anonymously, and data were matched to questionnaires using unique IDs to preserve confidentiality. Each sample was assigned a unique, randomly generated identification number to eliminate analysis bias. Simultaneously, questionnaire data corresponding to each participant were recorded. The samples were then centrifuged at 4000× *g* for 8 min at 4 °C. Following centrifugation, the resulting pellet was resuspended in 10 mL of sterile normal saline buffer and stored at −80 °C for subsequent DNA purification [18].

### 2.3. Salivary DNA Isolation and HPV Genotyping Protocol

Before nucleic acid extraction, the samples (1 mL each) were centrifuged for 15 min at 15,000× *g*. The resulting pellet was resuspended in 200 µL of lysis buffer. Nucleic acid isolation was performed using the Applied Biosystems MagMAX™ Viral/Pathogen II kit for automated extraction with the KingFisher™ Flex instrument (Applied Biosystems, Waltham, MA, USA). Following nucleic acid extraction, DNA concentration was quantified using a NanoDrop One spectrophotometer (Thermo Scientific, Waltham, MA, USA), and 50 ng of salivary DNA was used per PCR reaction. For genotyping, sample amplification was carried out by RT-PCR using the Allplex™ HPV28 kit (Seegene Inc., Seoul, South Korea), according to the manufacturer’s instructions. This multiplex assay allows the simultaneous amplification of target DNA from 14 high-risk human papillomavirus (HPV) types (16, 18, 31, 33, 35, 39, 45, 51, 52, 56, 58, 59, 66, 68, 26, 53, 69, 73, 82) and 9 low-risk HPV types (6, 11, 40, 42, 43, 44, 54, 61, 70). DNA purity was assessed by measuring the absorbance ratio at 260/280 nm using a NanoDrop One spectrophotometer (Thermo Scientific), with acceptable values ranging between 1.8 and 2.2. β-actin was used as an internal control to verify amplification integrity. All reactions were performed in an Eppendorf thermal cycler under the following conditions: initial denaturation at 95 °C for 8 min, followed by 35 cycles of 95 °C for 45 s, 56 °C for 30 s, and 72 °C for 30 s, with a final extension at 72 °C for 7 min [38,39].

### 2.4. Statistical Analysis

A structured statistical approach was utilized to assess the relationship between demographic characteristics, lifestyle factors, and awareness levels in relation to HPV vaccination status. Descriptive statistical methods were initially applied to summarize the dataset, including frequency distributions, means, and standard deviations. Correlation analysis was conducted to examine potential associations between key variables, while categorical variables were analyzed using cross-tabulations. The correlation coefficient “r” refers to Pearson correlation. As recommended, we performed a Shapiro–Wilk normality test on the numerical variables prior to applying Pearson correlation. The results confirmed a normal distribution (*p* > 0.05), supporting the use of parametric correlation methods. Percentages were calculated to identify patterns related to smoking status, awareness levels, and HPV vaccination uptake.

The data were initially entered and organized using Microsoft Excel, which served as the primary platform for data entry and preliminary formatting. This step ensured a clean and structured dataset that could be seamlessly imported into statistical software for further analysis.

To determine the predictors of HPV vaccination, a logistic regression model was implemented, incorporating independent variables such as age, sex, smoking status, and awareness of HPV-related risks. The regression model outputs, including coefficients, standard errors, *p*-values, and confidence intervals, were examined to assess statistical significance.

To facilitate data interpretation, graphical representations such as histograms, stacked bar charts, and a correlation heatmap were generated. All statistical analyses and tests were conducted using Python (version 3.11), employing libraries for data processing, visualization, and regression modeling, including Matplotlib (version 3.7.1). Additionally, MedCalc Statistical Software (version 22.013) was used for result validation, ensuring both accuracy and reproducibility. All statistical tests were two-tailed, with a significance threshold set at *p* < 0.05. No adjustments for multiple comparisons were made, given the exploratory nature of the study.

This rigorous statistical approach provided a robust analytical framework for evaluating factors associated with HPV vaccination status, enabling a comprehensive understanding of the variables influencing vaccination uptake.

## 3. Results

### 3.1. Participant Demographics and Health Status

The study included 199 participants, with an average age of 23.5 (±2.6) years. The majority of respondents were female, accounting for 79.4% of the sample, while males represented 20.6%. The distribution of ages was concentrated primarily in the early-to-mid-twenties, with some variability among participants. A small proportion (4%) of participants reported experiencing current health issues. Chronic conditions such as heart disease, lung disease, kidney disease, diabetes, anemia, or neurological disorders were noted in 5% of cases. Additionally, 10.6% of participants reported known allergies to food or medication, while 2.5% indicated previous severe adverse reactions to vaccinations (Table 1).

### 3.2. Smoking Behavior and Medical Background

Among the participants, 31.2% reported current smoking behavior. When analyzed by gender, smoking was more prevalent among male students (45.7%) compared to female students (25.4%), indicating a gender-related difference in tobacco use patterns within the study population.

The use of immunosuppressive therapy was absent among all participants, as no individuals reported taking medications such as cortisone, prednisone, or other steroids within the past three months. Additionally, no respondents had received a blood transfusion in the past year. None of the female participants were pregnant or breastfeeding at the time of the survey.

### 3.3. HPV Vaccination Rates and Awareness

**Figure 1:**
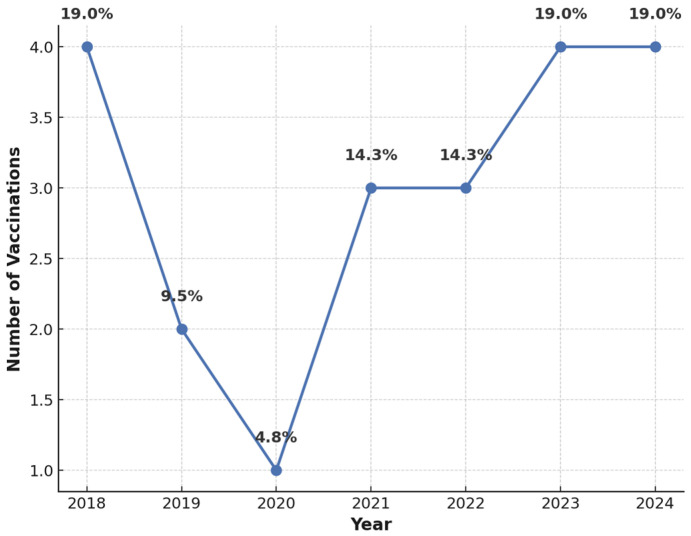
Histogram depicting HPV vaccination uptake by year.

The analysis of HPV vaccination rates revealed a relatively low uptake, with only 10.6% of participants having received the HPV vaccine. Among vaccinated individuals, immunization predominantly took place in 2023 and 2024, suggesting increased awareness in recent years (Figure 1). Despite low vaccination rates, 96.9% of participants acknowledged the safety and efficacy of HPV vaccination in cancer prevention. Furthermore, 94.5% believed that vaccinated individuals could no longer transmit the virus within the community.

### 3.4. Age and Gender Differences in Awareness and Vaccination

Salivary screening revealed that 10% of the participants tested positive for HPV types, underscoring the potential utility of saliva-based diagnostics as a non-invasive tool for early detection of HPV infections among asymptomatic young adults.

Among the participants, HPV vaccination uptake was low, with only 10.6% reporting having received the vaccine. When disaggregated by gender, 8.5% of the total sample were vaccinated females, while only 2.0% were vaccinated males. This gender-based difference was statistically significant (*p* = 0.0123), indicating that female students were more likely to be vaccinated.

Regarding smoking habits, a significant difference was observed (*p* = 0.0456), with the smoking rate among males (45.7%) being significantly higher than that recorded among females (25.4%). HPV awareness, however, did not differ significantly between genders (*p* = 0.0789), although slightly more females (88.1%) were aware of HPV vaccination compared to males (84.3%) (Table 2).

When analyzing vaccination rates by age, participants were categorized into two groups: those under 25 years old and those 25 years or older. A significant association was found between age group and vaccination status (*p* = 0.0312), with younger students (<25 years) having a higher vaccination rate (68.4%) compared to 50.0% among older students. Smoking rates did not differ significantly between age groups (*p* = 0.0678), with younger students reporting a 29.5% smoking rate compared to 34.8% among older students.

A statistically significant difference in HPV awareness levels was observed between age groups (*p* = 0.0234), with 89.6% of younger students demonstrating awareness compared to 72.4% of older students (Table 3).

### 3.5. Family History and Wart Incidence

Among the surveyed participants, 31.7% reported having a family history of cancer. Of these, various cancer types were identified, including breast, colon, cervical, and pulmonary cancers, among others. Among participants with a family history of cancer (representing 31.7% of the sample), only 15.9% of this subgroup reported having received the HPV vaccine. This indicates that an elevated familial cancer risk did not translate into increased vaccination uptake. Clarifying this distinction, we note that the 15.9% refers specifically to those with a family history of cancer, not the entire study population. Notably, just 15.9% of those with a family history of cancer were vaccinated, indicating no increase in uptake among higher-risk individuals. When analyzed by sex, 12.0% of female participants were vaccinated, compared to only 4.9% of males. This disparity highlights a potential gap in targeted preventive strategies, especially among men, and emphasizes the importance of tailored health education and vaccination efforts across gender lines.

A total of 16.6% of participants reported the presence of papillomas or warts, highlighting the relevance of early screening and preventive vaccination efforts. Among these individuals, the majority were female (78.8%), while males accounted for 21.2% of cases.

Among the 199 participants, 20 individuals (10%) tested positive for high-risk HPV strains (types 16 or 18) via salivary PCR analysis. Of these 20 HPV-positive individuals, 18 had not received any dose of the HPV vaccine. The remaining two positive cases were among vaccinated individuals, both of whom had received only one dose of the vaccine, administered within six months prior to sample collection.

**Figure 2:**
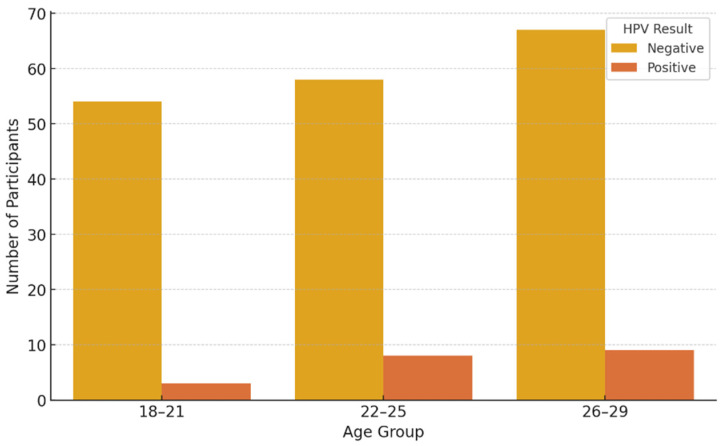
HPV PCR test results stratified by age group.

**Figure 3:**
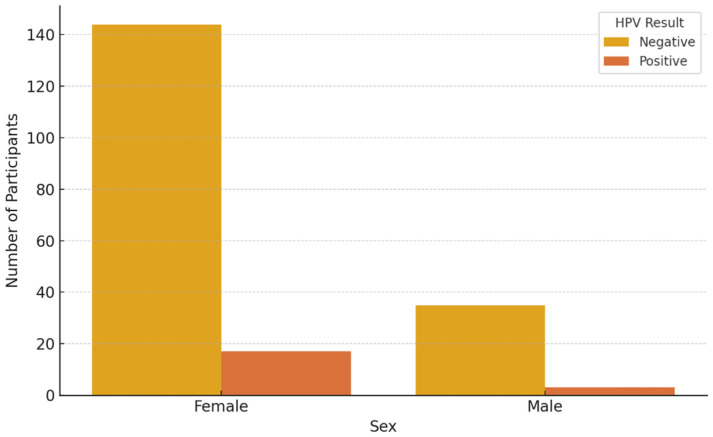
HPV PCR test results stratified by sex.

Figure 2 and Figure 3 present HPV PCR test results stratified by age and sex, respectively, showing demographic variation in viral detection rates.

**Figure 4:**
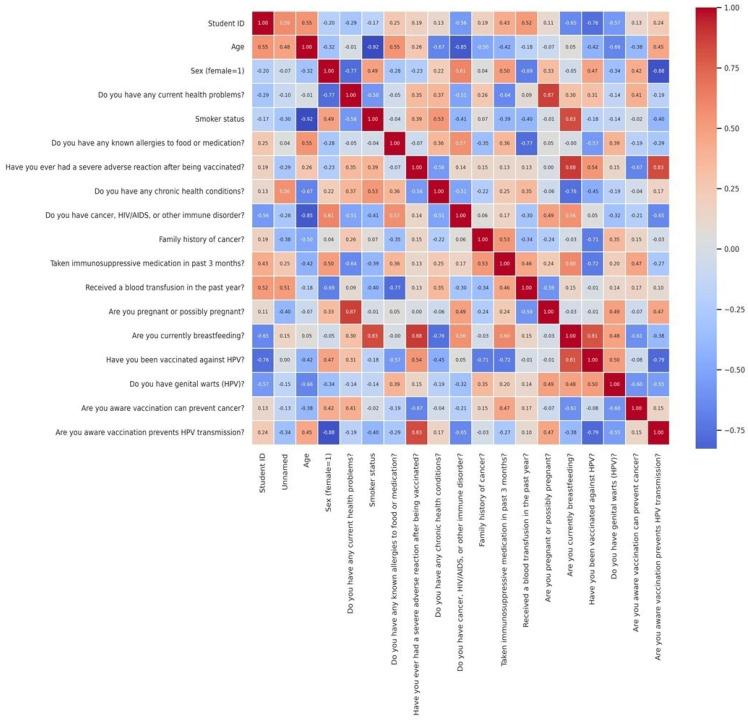
Exploratory correlation matrix illustrating associations between selected demographic, behavioral, and vaccination-related variables. Color intensity and numeric labels reflect Pearson correlation coefficients (r).

The correlation matrix provided insights into the degree of association between various factors influencing HPV vaccination status. While most variables exhibited weak correlations, notable patterns emerged in terms of lifestyle factors and vaccine uptake. A cross-tabulation analysis of smoking status and HPV vaccination suggested potential differences in vaccine uptake between smokers and non-smokers, likely reflecting variations in health awareness, risk perception, or healthcare-seeking behaviors. The correlation analysis revealed predominantly weak to moderate relationships among the investigated variables related to health status and HPV vaccination knowledge. A moderate positive correlation was identified between awareness that HPV vaccination can prevent cancer and the understanding that it reduces viral transmission (r = 0.52). Furthermore, the presence of chronic health conditions was moderately associated with recent use of immunosuppressive medication (r = 0.47) and with a history of blood transfusion (r = 0.43), indicating that these variables co-occurred more frequently within the same individuals. Conversely, demographic variables such as age and sex demonstrated weak correlations with key outcome variables, including HPV vaccination status (r = 0.12 for age, r = −0.08 for sex), indicating limited predictive value in this sample. The correlation between having been vaccinated against HPV and the presence of genital warts was also low (r = −0.15) (Figure 4).

While the correlation matrix provides insight into potential associations, it is important to note that most correlations were weak and did not reach statistical significance. The matrix was included as an exploratory tool to visualize overall trends, not to infer causality.

The logistic regression analysis identified several significant predictors of HPV vaccination status. Smoking status was the strongest positive predictor (β = 1.49, OR = 4.44, *p* = 0.0045), indicating that smokers were over four times more likely to be vaccinated compared to non-smokers. Belief in the safety and cancer-preventive efficacy of the HPV vaccine was also positively associated with vaccine uptake (β = 1.29, OR = 3.63, *p* < 0.05). Female sex showed a moderate association (β = 1.01, OR = 2.74), suggesting a trend toward higher vaccination rates among women, though this did not reach statistical significance. Age demonstrated a minimal effect (β = 0.07, OR = 1.07, *p* > 0.05), indicating that it was not a meaningful predictor in this model. Interestingly, belief that HPV vaccination reduces community-level viral transmission was negatively associated with vaccination status (β = −0.82, OR = 0.44). The model intercept was negative (β = −2.12), reflecting a generally low baseline probability of vaccination in the absence of these influencing factors.

Participant perceptions of HPV vaccination were examined in relation to three key domains: beliefs about vaccine safety and efficacy, perceptions regarding the vaccine’s impact on viral transmission, and intentions for future vaccination.

When asked whether they believed that HPV vaccination was safe and effective in preventing cancer, 76.2% of participants responded affirmatively, while 13.2% disagreed and 10.6% were uncertain. Stratified by sex, 81.0% of female respondents believed in the vaccine’s safety and efficacy, compared to 61.0% of males. Meanwhile, 10.1% of females and 24.4% of males responded “no”, and 8.9% of females versus 14.6% of males indicated uncertainty. These findings suggest greater confidence in HPV vaccination among women, whereas male participants exhibited a higher degree of skepticism or lack of awareness.

A similar pattern emerged when participants were asked whether they believed that vaccinated individuals could no longer transmit HPV within the community. In total, 79.4% responded “yes”, 13.1% said “no”, and 7.5% were unsure. Among females, 82.9% believed vaccination prevents transmission, compared to 65.9% of males. Conversely, 10.8% of females and 22.0% of males disagreed, while 6.3% and 12.2%, respectively, were unsure. Although general awareness of HPV vaccine benefits was high, these results underscore persistent sex-based differences in perception, with men showing more doubt or misinformation.

Regarding future vaccination intent, 49.2% of all participants expressed willingness to receive the HPV vaccine, 12.6% reported no intention, and 27.1% were uncertain. Additionally, 10.6% stated that they had already been vaccinated. Among women, 48.1% intended to receive the vaccine, 11.4% did not, and 27.8% were unsure. Among men, 53.7% expressed intent, 17.1% declined, and 24.4% were undecided. While intention to vaccinate appears relatively strong—especially among men—the considerable proportion of undecided participants, together with the observed inconsistencies, highlights the need for clearer communication and targeted educational efforts to support informed decision-making.

These results are summarized in Table 4, which presents HPV PCR results, vaccination status, and perceptions regarding vaccine safety, efficacy, and transmission prevention stratified by gender.

## 4. Discussion

The rising prevalence of HPV-positive oral cancers, along with the shifting demographic patterns of affected individuals, highlights the growing importance of preventive measures within dental practice. Dentists are uniquely positioned to contribute to the early identification and prevention of HPV-related oral malignancies through patient education, risk assessment, and routine screening for (pre)malignant lesions. Addressing topics such as HPV transmission and safe sexual practices can play a key role in curbing the increasing trend of HPV-associated oral cancers.

Given their evolving role as future oral health professionals, dental students represent a strategically significant population for targeted HPV prevention initiatives. Their academic training extends beyond the oral cavity to encompass broader medical knowledge, including systemic diseases, immune status, and behavioral risk factors. This positions them uniquely to provide holistic health education, addressing HPV transmission, vaccine benefits, and its association with both oropharyngeal and other HPV-related malignancies. As emphasized by Lipsky et al., dental professionals are increasingly recognized as key stakeholders in HPV-related cancer prevention—not only through clinical screenings, but also via routine patient interactions that offer opportunities for counseling and public health guidance [40]. Assessing students during their formative academic years underscores the urgency of integrating comprehensive HPV education and communication training into dental curricula, empowering them to become proactive advocates for vaccination and early detection across healthcare settings.

However, multiple studies have shown that healthcare students, including those in dental and medical fields, often possess limited knowledge regarding oral cancer and its risk factors, indicating a clear need for improved educational strategies in this area.

Our study focused on screening the saliva of healthy dental students aged 18–25 from the Victor Babeș University of Medicine and Pharmacy in Timișoara for the presence of HPV. Additionally, it aimed to raise awareness among participants regarding the role of HPV 16 and 18 subtypes in oral cancers, as well as the benefits of vaccination, which is strongly recommended for this age group. In our study, the vast majority of dental students (96.9%) acknowledged the safety and efficacy of the HPV vaccine, and 94.5% believed that vaccinated individuals could no longer transmit the virus. Despite this high level of awareness, actual vaccination uptake remained low, with only 10.6% of participants reporting having received the HPV vaccine. Notably, vaccination rates were significantly higher among younger students and females.

Although general awareness regarding HPV and its link to cancer was relatively high, the gap between knowledge and preventive behavior underscores the presence of external barriers, such as accessibility, vaccine hesitancy, or insufficient emphasis on vaccination in the dental curriculum. These findings align with prior research highlighting poor vaccination rates among dental students despite moderate to high levels of awareness [41].

Additionally, the moderate associations between chronic health conditions, recent use of immunosuppressive medications, and history of blood transfusions may reflect a clustering of health vulnerability indicators. These overlaps suggest the presence of a medically at-risk subgroup within the student population, warranting closer attention in preventive strategies and health education programs.

Reducing tobacco and alcohol consumption, along with maintaining a healthy diet, may lower the risk of oral HPV infections [36,42]. The development of HPV vaccines has brought significant hope for the prevention of related cancers. However, there is still a concerning lack of awareness, even among medical students. The level of awareness regarding HPV infection and vaccination among dental students in our study, although relatively high in terms of general knowledge, did not translate into equally high vaccination uptake. Only 10.6% of participants had received the HPV vaccine, a rate comparable to previous findings among healthcare students in regions such as Malaysia (3.6%) and Hong Kong (13.3%) [36,42]. Despite nearly all students acknowledging the efficacy and safety of the vaccine, a notable gap persists between knowledge and behavior, suggesting that informational awareness alone is not sufficient to drive vaccination. This may reflect structural or perceptual barriers such as limited access to vaccination services, concerns about side effects, or sociocultural hesitation related to HPV’s transmission route. Similarly to findings from other studies [13,43], our results demonstrated a significant association between academic year and HPV awareness, with senior students displaying higher levels of knowledge, likely due to greater exposure to clinical training and curricula addressing cancer prevention, reflect a consistent pattern of health literacy in this subgroup, particularly regarding the understanding of HPV vaccination as a preventive measure against both viral transmission and cancer development.

Interestingly, while no significant gender-based difference was observed in HPV knowledge, female students showed higher vaccination rates, echoing trends reported in other populations [44]. Beyond vaccination uptake observed among female participants, the data also indicated a greater frequency of self-reported wart presence in this subgroup. While this may suggest potential biological differences in HPV manifestation between sexes, it is equally plausible that the disparity reflects gender-specific patterns in symptom perception, health literacy, or the propensity to seek medical evaluation [45]. The results of the current study resonate with prior multinational data indicating that while knowledge of HPV and its link to oropharyngeal cancers is variable, many dental students express a willingness to receive training and expand their understanding. In the study by Lingam et al., Indian students displayed the highest awareness levels, likely due to their increased clinical exposure to HPV-related conditions, as India holds one of the highest global burdens of oral cancers. Notably, female students across all surveyed countries demonstrated more favorable attitudes toward HPV vaccination and were more comfortable discussing HPV-related topics with patients compared to males, a finding attributed in part to greater awareness about HPV’s role in cervical cancer and the influence of gendered communication dynamics in healthcare [10]. Despite a generally high level of awareness regarding the benefits of HPV vaccination, our findings highlight persistent sex-based disparities in perception and confidence, with male participants demonstrating higher levels of uncertainty or susceptibility to misinformation.

Previous studies from countries such as Saudi Arabia and Malaysia have shown that students commonly receive information about HPV through formal education, the internet, and media sources—highlighting the influence of multiple channels in shaping awareness [36,44].

Our study findings align with previous Romanian research in recognizing a strong correlation between students’ academic level and their awareness of HPV and related health risks. For instance, Murariu et al. [46] reported that fifth-year dental students and residents had significantly greater knowledge of oral cancer and HPV infection compared to fourth-year students, primarily due to curriculum exposure during the clinical years. Similarly, our results show that senior dental students had higher awareness and vaccination rates than junior students, reinforcing the importance of curricular timing in shaping preventive health knowledge. Consistent with our findings, previous research among Spanish dental students showed that clinical-level students demonstrated significantly higher levels of knowledge regarding the HPV–oropharyngeal cancer link and HPV vaccination compared to preclinical peers. In that study, clinical students outperformed in 12 out of 16 knowledge items related to oral HPV, and in all vaccination-related items, with statistically significant differences across the board. This reinforces the argument that exposure to clinical disciplines like oral pathology and public health is critical for building competent future practitioners who are capable of promoting HPV prevention [12].

While Murariu et al. [46] found that 81.8% to 82.5% of students acknowledged the existence of the HPV vaccine, only 23.4% to 34.9% believed it could prevent oral cancer, indicating a significant knowledge gap regarding HPV’s oncogenic potential beyond cervical cancer. In our study, despite high levels of general awareness (over 96% recognized the cancer-preventive benefit of the vaccine), actual vaccination uptake was notably lower (10.6%). This mismatch between knowledge and action reflects similar challenges in translating awareness into preventive behavior and may be influenced by accessibility, cultural perceptions, or lack of institutional emphasis on vaccination. The progressive development and approval of HPV vaccines have played a critical role in reducing the global burden of HPV-related diseases, particularly cervical cancer. Since 2007, bivalent and quadrivalent vaccines have been available in Europe, targeting HPV types 16 and 18, which are responsible for approximately 70% of cervical cancer cases [47,48]. These formulations also provide partial cross-protection against other high-risk types such as HPV 31, 33, and 45. Moreover, the quadrivalent vaccine extends protection to low-risk HPV types 6 and 11, which are linked to 90% of benign genital warts and cases of recurrent respiratory papillomatosis. In an effort to increase vaccine uptake and compliance, the originally recommended three-dose schedule was reduced to two doses for adolescents in 2014. The approval of the nonavalent vaccine by the European Medicines Agency in 2015, which covers five additional oncogenic HPV types (31, 33, 45, 52, and 58), has further expanded protection, raising the potential prevention of cervical cancer from approximately 70% to 90% [17].

Moreover, this discrepancy between high knowledge and low vaccine uptake may also reflect the influence of misinformation or insufficient confidence in specific vaccine benefits, particularly in relation to oropharyngeal cancer prevention.

Despite being future healthcare providers, many medical students in Romania exhibit incomplete knowledge and varying degrees of HPV vaccine hesitancy. Prior studies have shown that awareness alone may not translate into vaccine acceptance. Psychological and motivational factors often override factual understanding. For example, while Voidăzan et al. (2016) found relatively high awareness of HPV among Romanian medical students, actual understanding and vaccine uptake remained low [27]. Similarly, Grigore et al. (2018) reported that fewer than 40% of surveyed Romanian women had been vaccinated, citing low awareness and limited confidence in vaccine efficacy [49]. These findings highlight a crucial need for tailored educational and behavioral strategies that address not only informational gaps but also emotional and trust-related barriers.

Comparatively, the study by Dincă et al. (2024) [50] emphasized dental students’ gaps in understanding vaccine-preventable diseases (VPDs), with only 45.1% correctly identifying HPV as vaccine-preventable. Our cohort showed higher HPV awareness, suggesting potential differences in institutional focus or educational strategies. However, similar to Dincă et al. [50], we observed that younger students and those earlier in their education demonstrated less accurate knowledge about HPV, underlining a systemic need for earlier and more comprehensive integration of HPV-related content in dental curricula. Furthermore, while Dincă et al. noted reluctance among students to treat patients with chronic viral infections like HIV due to high perceived risk. This indicates that, despite theoretical knowledge, there may be a lack of preparedness for patient communication on sensitive topics—an area requiring attention through practical training and role-play scenarios during clinical education. One limitation of this study is the low representation of male participants, who accounted for only 20.6% of the sample. This gender imbalance reflects the actual demographic composition of the dental student population at our institution during the time of data collection, where female enrollment was substantially higher. However, it may limit the generalizability of findings related to sex-based differences in HPV knowledge, attitudes, and vaccine uptake. The underrepresentation of male perspectives could introduce bias, especially when interpreting male-specific barriers to vaccination or health communication patterns. Future research should aim to include more balanced samples to validate and expand these findings. This underlines the critical role of universities in structuring effective educational interventions. However, the relatively low self-reported intention to vaccinate among students, despite high awareness, signals a need for targeted educational programs that not only inform but also actively address misconceptions and barriers to vaccine uptake. Moreover, although the overall intention to vaccinate appeared relatively favorable, particularly among male participants, a substantial proportion of respondents remained undecided. This hesitation, when viewed alongside the observed discrepancies between HPV-related knowledge and actual vaccination behavior, underscores the necessity for more nuanced communication strategies and the development of evidence-informed educational interventions to support informed and autonomous decision-making among future dental professionals. Reinforcing HPV-related content in the preclinical dental curriculum, coupled with stronger recommendations from healthcare providers, may enhance students’ readiness to both receive and advocate for vaccination. This is especially relevant as dental professionals increasingly become key figures in the early detection and prevention of HPV-related oropharyngeal cancers.

However, the contrast between high awareness and low vaccination uptake suggests the presence of external barriers such as limited accessibility, concerns about potential side effects, or misinformation contributing to vaccine hesitancy.

An important aspect that emerged from our findings is the potential role dental professionals can play in HPV-related cancer prevention, particularly through patient education and early detection strategies. However, despite the acknowledged importance of HPV prevention, students’ confidence in discussing HPV-related topics with patients remains an area requiring further development. This hesitancy, often observed in culturally conservative contexts, may be influenced by discomfort in addressing sexually transmitted infections or fear of being perceived as overstepping professional boundaries. Previous studies have reported similar trends, where dental students expressed reluctance in recommending HPV vaccination, especially to patients of the opposite sex [13,51]. Despite growing evidence of the preventive role that dentists can play in HPV-related cancer control, substantial gaps remain in formal education on this topic. A national survey of U.S. dental schools revealed that while 85% include HPV-related content in required courses, only 10% address prevention strategies and a mere 11% provide training in patient education on HPV. This suggests that educational efforts are still largely theoretical and may not equip students with the practical skills necessary for effective HPV prevention in clinical settings [8]. While most dental students agree that educating patients about the HPV–oropharyngeal cancer link falls within their professional responsibilities, many lack the confidence to engage in such conversations. In a recent U.S. study, 44% of students reported anticipating discomfort when recommending the HPV vaccine, and only 33.9% felt “very confident” discussing HPV risk factors with patients [8]. These findings underscore a critical need for communication training, particularly in discussing sexually transmitted infections in culturally sensitive environments [8]. Participant hesitations toward HPV vaccination, as reflected in their uncertainty or misconceptions regarding vaccine safety and transmission prevention, highlight the need for targeted educational interventions. While outright refusal was rare, approximately 27.1% of participants reported indecision about future vaccination, and 13.2% expressed doubts about vaccine safety or efficacy. Moreover, a lower proportion (79.4%) believed that the vaccine prevents HPV transmission, despite widespread acknowledgment of its cancer-preventive role. Gender-based differences were also notable—male students were more likely to express hesitation, with 24.4% either disagreeing or unsure about the vaccine’s safety, compared to 10.1% of female respondents. These findings suggest that hesitancy may be driven more by informational gaps and trust issues than by active opposition and point to the importance of addressing these perceptual barriers through targeted, gender-sensitive communication strategies. The absence of clear clinical protocols for oral cancer screening was also identified as a significant barrier among dental students. A Dutch study found that students strongly supported the development of standardized screening procedures and emphasized the need for additional training in clinical oral examinations [13]. The establishment of such protocols could enhance consistency and confidence in early detection practices [13]. Similarly to findings in Romania, Dutch dental students reported low self-confidence in conducting oral cancer examinations. When asked to rate their confidence, visual inspection received an average score of 3.2 out of 4 (where 4 indicates “not confident”), and manual palpation scored even lower. These self-assessments highlight a universal need for improved clinical training and early exposure to head and neck cancer diagnostics during dental education [13].

Given the increasing burden of HPV-related oropharyngeal cancers and the regular patient contact dentists have, it is essential to integrate structured communication training within the dental curriculum to improve future professionals’ confidence and competence in addressing sensitive health topics.

In parallel, our study supports the emerging role of saliva as a non-invasive diagnostic tool for HPV detection. The ease of collection, patient comfort, and the molecular content of saliva—ranging from DNA to exosomes—make it an appealing medium for early identification of high-risk HPV strains associated with oropharyngeal cancers. Our protocol, which involved pre-analytical standardization and PCR-based genotyping, highlights the feasibility of integrating salivary diagnostics into clinical and educational settings. As such, future dental professionals should be made aware of these advancements, which not only support preventive oncology but also align with the broader move toward personalized and minimally invasive medicine. Expanding the role of dentists in HPV surveillance and prevention, through both education and clinical screening, could significantly contribute to global efforts in reducing HPV-related cancer incidence. While most HPV-related research in Romania has focused on cervical cancer, our findings highlight the growing need to address oropharyngeal cancers within dental medicine. Dentists, due to their regular contact with patients, are well-positioned to contribute to HPV education and early detection. Romanian dental education should therefore include targeted communication training and practical prevention strategies. As emphasized by Diaconescu et al. (2021), psychological factors—such as health beliefs and stage of academic training—play a significant role in vaccination intent, reinforcing the value of tailored interventions during dental education [28]. Future research should investigate the integration of salivary HPV screening into dental practice, particularly for high-risk groups. Since the participating dental students were still in training and not yet practicing independently, the main objective of our study was to evaluate their opinions and attitudes toward discussing HPV-related topics with future patients. This focus allowed us to explore their perceived role in HPV prevention, even in the absence of direct clinical responsibility.

In our study, salivary screening was performed on a subset of participants who consented to molecular testing for HPV. The results revealed a low prevalence of detectable HPV DNA in saliva samples, aligning with the self-reported low rate of HPV-related oral lesions and limited vaccination uptake. These findings are consistent with those of Fakhry et al. (2011), who reported that the detection of oral HPV DNA in asymptomatic individuals using salivary samples remains relatively rare, particularly in young populations with no known risk factors [52]. Despite its low sensitivity in general populations, salivary screening remains a promising non-invasive tool for early detection, especially in high-risk groups or clinical settings where visual inspection alone may be insufficient.

Since our study had a small sample size of 199 participants, specifically dental students from the Victor Babeș University of Medicine and Pharmacy in Timișoara, it may reflect a sampling bias and may not provide a complete picture of HPV infection among the general population. However, this research has undoubtedly contributed to raising awareness among the participants, as reflected in our findings. The increased knowledge about HPV and its associated health risks has led to a greater understanding among these young adults of the importance of timely vaccination and its acceptance. This represents a crucial step in the right direction, especially considering that a previous questionnaire-based study in Romania indicated a low acceptance rate of the HPV vaccine. Given the significance of HPV-related diseases, enhancing awareness and promoting vaccination among future healthcare professionals is both an urgent necessity and a long-term investment in public health. In our study, all data regarding HPV awareness, vaccination status, and sexual health perceptions were self-reported, which introduces the potential for social desirability bias and underreporting of sensitive behaviors. The cross-sectional design does not allow for establishing causal relationships between variables such as awareness and vaccine uptake. Finally, although saliva-based HPV detection provides a non-invasive screening approach, the study did not assess participants’ oral health status or correlate positivity with clinical symptoms, which could have further validated the findings.

One of the key takeaways from this study is the use of a non-invasive screening method to detect the presence of HPV 16 and 18, making early identification more accessible. The study specifically focuses on young adults, a demographic that needs increased awareness regarding timely protection against HPV-related diseases. Furthermore, this research highlights the necessity of incorporating HPV immunization into public healthcare policies to mitigate the long-term burden of HPV-related illnesses. Future research should include larger, multicentric samples across Romanian universities, assess long-term behavior change following educational interventions, and explore patient perspectives on receiving HPV-related advice from dentists. A crucial message conveyed is that there is a significant gap in knowledge regarding HPV-related oral cancer and its vaccine, an issue that also applies to Romania. This underlines the need for increased awareness campaigns and improved vaccination strategies to enhance public health outcomes.

## 5. Conclusions

This study highlights critical gaps between knowledge, attitude, and actual HPV vaccination behavior among Romanian dental students. While the majority of participants demonstrated a high level of awareness regarding the risks associated with HPV and the benefits of vaccination, the overall vaccination rate remained low. Factors such as academic level, age, gender, and smoking status were significantly associated with awareness and vaccine uptake, underscoring the need for targeted educational interventions. Furthermore, the integration of saliva-based diagnostics shows promising potential for early, non-invasive detection of HPV-related malignancies, particularly oropharyngeal cancers. Strengthening HPV-related content within the dental curriculum, along with practical training in patient communication and preventive strategies, may empower future dental professionals to play a more active role in public health efforts aimed at reducing the burden of HPV-related cancers.

## Figures and Tables

**Table 1:** Background characteristics of study participants (N = 199).

Characteristic	Value
Total participants	199
Average age (years)	23.5 ± 2.6
Female participants (%)	79.4%
Male participants (%)	20.6%
Smokers (%)	31.2%
Non-smokers (%)	68.8%
Participants with chronic health conditions (%)	5%
Participants with food/medication allergies (%)	10.6%
Participants with past adverse vaccine reactions (%)	2.5%
Participants with family history of cancer (%)	31.7%
Participants reporting papillomas or warts (%)	16.6%

**Table 2:** Gender-based differences in smoking and HPV vaccination indicators.

Characteristic	Males (%)	Females (%)	*p*
Smokers	45.7%	25.4%	0.045
HPV vaccine awarness	84.3%	88.1%	0.078
HPV vaccination rates	52.1%	65.2%	0.12

**Table 3:** Age-based differences in HPV vaccine awareness, uptake, and smoking behavior.

Characteristic	<25 Years (%)	≥25 Years (%)	Significant Difference (*p*)
Vaccination Rate	68.4%	50%	0.0312
Smoking Rate	29.5%	34.8%	0.0678
HPV vaccine awareness	89.6%	72.4%	0.0234

**Table 4:** Gender-stratified distribution of HPV status, vaccination uptake, and related perceptions among dental students.

Indicator	Females (N = 158)	Males (N = 41)
HPV Positive	9.5% (15)	12.2% (5)
HPV Negative	90.5% (143)	87.8% (36)
Belief in vaccine safety and efficacy	81.0% (128)	61.0% (25)
Belief vaccine prevents transmission	82.9% (131)	65.9% (27)
Intends to vaccinate in future	48.1% (76)	53.7% (22)
Already vaccinated	10.6% (17)	10.6% (4)
Does not intend to vaccinate	11.4% (18)	17.1% (7)
Unsure about vaccination	27.8% (44)	24.4% (10)

## Data Availability

The data presented in this study are available on request from the corresponding author.

## Abbreviations

The following abbreviations are used in this manuscript:

HPV	Human papilloma virus
DNA	Deoxyribonucleic acid
